# Complete genome sequence of *Solobacterium moorei* JCM 10645^T^ isolated from a human stool sample

**DOI:** 10.1128/MRA.00965-23

**Published:** 2023-11-29

**Authors:** Kota Oshibuchi, Jiayue Yang, Nozomu Obana, Shinji Fukuda, Kazuharu Arakawa

**Affiliations:** 1 Institute for Advanced Biosciences, Keio University, Tsuruoka, Yamagata, Japan; 2 Systems Biology Program, Graduate School of Media and Governance, Keio University, Fujisawa, Kanagawa, Japan; 3 MIRAI Technology Institute Yokohama, Yokohama, Japan; 4 Transborder Medical Research Center, Institute of Medicine, University of Tsukuba, Tsukuba, Ibaraki, Japan; 5 Gut Environmental Design Group, Kanagawa Institute of Industrial Science and Technology, Kawasaki, Kanagawa, Japan; 6 Laboratory for Regenerative Microbiology, Juntendo University Graduate School of Medicine, Bunkyo-ku, Tokyo, Japan; 7 Faculty of Environment and Information Studies, Keio University, Fujisawa, Kanagawa, Japan; Rochester Institute of Technology, Rochester, New York, USA

**Keywords:** *Solobacterium moorei*, complete genome, nanopore sequencing, genome assembly

## Abstract

*Solobacterium moorei* JCM 10645^T^ is an obligately anaerobic Gram-positive bacterium that was isolated from a human stool sample, generally known as a bacterium associated with sepsis, bacteremia, halitosis, and periodontal disease. In this study, we report the complete genome sequence of this strain, which is 2.615 Mbp with a 37.2% GC content.

## ANNOUNCEMENT

*Solobacterium moorei* JCM 10645^T^ is a Gram-positive, bacilliform, non-spore-forming, and obligately anaerobic bacterium that has been isolated from a human stool sample by Kageyama et al. (1). This bacterium is the type strain of *Solobacterium moorei*. This species is generally known as a bacterium associated with sepsis, bacteremia, halitosis, and periodontal disease (2). Here, we analyzed the complete genome sequence of *Solobacterium moorei* JCM 10645^T^.

*Solobacterium moorei* JCM 10645^T^, obtained from the Japan Collection of Microorganisms, was cultured in GAM medium at 37°C in an anaerobic condition for 24 hours. The genomic DNA was extracted from the harvested cells using Genomic-tip 20/G (Qiagen) according to the manufacturer’s protocol for both long- and short-read sequencing. A long-read sequencing library was prepared using the Rapid Barcoding Sequencing Kit (SQK-RBK004) (Oxford Nanopore Technologies) and sequenced using an R10.3 Flowcell (FLO-MIN111) on a GridION X5 device (GridION software release 21.05.25; Oxford Nanopore Technologies). Base calling (Super Accurate Mode), demultiplexing, and adapter removal were performed using Guppy v.6.3.8 software. Assembly was performed using all sequenced long reads (641.7 Mb, 227,408 reads in total, N50: 4.643 kb) by Canu version 2.2 (3). Short-read sequencing was performed for error correction using the HyperPlus library preparation kit (Kapa Biosystems) and a MiSeq sequencer (Illumina) with Reagent Kit v3 600 cycles as 300 bp paired ends (Illumina). Using filtered reads by fastp tool (https://github.com/OpenGene/fastp; 4.6M out of 9.7M paired reads), error correction was then performed by aligning the reads using Burrows-Wheeler Aligner v.0.7.11 (4), and subsequently, the polished sequence was then generated using Pilon v.1.23 (5). Assembly quality was assessed using CheckM (6) on the DFAST server leading to a predicted completeness score of 100% (Erysipelotrichaceae marker lineage). The genome was annotated using the DDBJ Fast Annotation and Submission Tool (DFAST) version 1.2.18 (7). All software was used with default settings unless otherwise specified.

The assembled genome consists of one linear chromosome of 2,615,268 bp with a GC content of 37.2%, harboring 2,599 protein-coding genes, 6 rRNAs, and 43 tRNAs. Volatile sulfur compound production has been reported for this species (8), and BLASTP search for the hydrogen-sulfide-producing enzyme MegL (SwissProt: MEGL_FUSNN) (9) identified a potential homolog by bi-directional identity (e = 2e-103). The availability of the genome resource can potentially contribute to the molecular mechanisms of the association of *Solobacterium moorei* in halitosis.

## Data Availability

The chromosome sequence reported here was deposited in DDBJ under the accession number AP028934, and the raw reads were deposited in the Sequence Read Archive (SRA) under the BioProject accession number PRJNA1013036 as SRR25909846 and SRR25909847 runs.
